# Dissecting *Escherichia coli* Outer Membrane Biogenesis Using Differential Proteomics

**DOI:** 10.1371/journal.pone.0100941

**Published:** 2014-06-26

**Authors:** Alessandra M. Martorana, Sara Motta, Dario Di Silvestre, Federica Falchi, Gianni Dehò, Pierluigi Mauri, Paola Sperandeo, Alessandra Polissi

**Affiliations:** 1 Dipartimento di Biotecnologie e Bioscienze, Università di Milano-Bicocca, Milan, Italy; 2 Istituto di Tecnologie Biomediche (ITB-CNR), Segrate, Milan, Italy; 3 Dipartimento di Bioscienze, Università degli Studi di Milano, Milan, Italy; University of Padova, Medical School, Italy

## Abstract

The cell envelope of Gram-negative bacteria is a complex multi-layered structure comprising an inner cytoplasmic membrane and an additional asymmetric lipid bilayer, the outer membrane, which functions as a selective permeability barrier and is essential for viability. Lipopolysaccharide, an essential glycolipid located in the outer leaflet of the outer membrane, greatly contributes to the peculiar properties exhibited by the outer membrane. This complex molecule is transported to the cell surface by a molecular machine composed of seven essential proteins LptABCDEFG that form a transenvelope complex and function as a single device. While advances in understanding the mechanisms that govern the biogenesis of the cell envelope have been recently made, only few studies are available on how bacterial cells respond to severe envelope biogenesis defects on a global scale. Here we report the use of differential proteomics based on Multidimensional Protein Identification Technology (MudPIT) to investigate how *Escherichia coli* cells respond to a block of lipopolysaccharide transport to the outer membrane. We analysed the envelope proteome of a *lptC* conditional mutant grown under permissive and non permissive conditions and identified 123 proteins whose level is modulated upon LptC depletion. Most such proteins belong to pathways implicated in cell envelope biogenesis, peptidoglycan remodelling, cell division and protein folding. Overall these data contribute to our understanding on how *E. coli* cells respond to LPS transport defects to restore outer membrane functionality.

## Introduction

**Figure 1:**
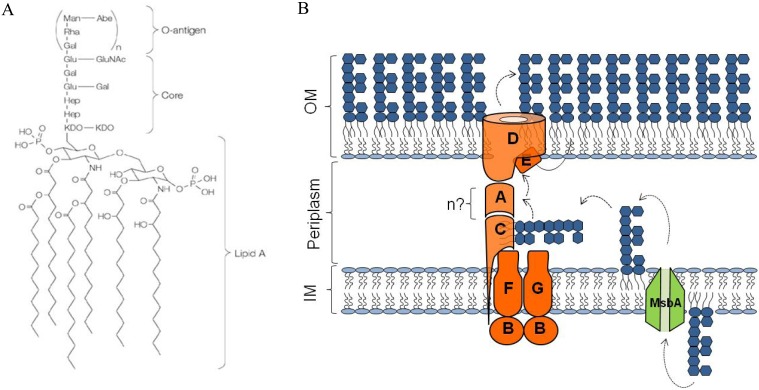
LPS structure and transport in *Escherichia coli*. A) Chemical structure of LPS. O-antigen is indicated in parenthesis as it is not synthesized in *E. coli* K12 derivatives. B) LPS transport from IM to OM. The MsbA protein catalyzes LPS flipping across the IM that is then exported to the cell surface by the Lpt machinery.

The outer membrane (OM) of Gram-negative bacteria [1] is an asymmetric membrane containing phospholipids and a unique glycolipid lipopolysaccharide (LPS) in the inner and outer leaflet, respectively [2]. OM proteins (OMPs) and lipoproteins are also embedded and anchored, respectively, in the OM [3]. LPS is a complex molecule that can be structurally divided in three elements: lipid A, the hydrophobic moiety that anchors LPS in the outer membrane, the core oligosaccharide and the *O*-antigen (Figure 1). The OM mainly serves as a protective barrier enabling Gram-negative bacteria to survive in harsh environments and to exclude several toxic molecules effective against Gram-positive organisms [1]. LPS mainly contributes to the OM permeability barrier properties as LPS molecules are tightly packed in the OM and form a very effective barrier against hydrophobic compounds [2]. Biosynthesis of LPS components occurs in the cytoplasm and at the cytoplasmic side of the inner membrane (IM). The core-lipid A moiety is first flipped by the essential ABC transporter MsbA across the IM [4], [5] ligated with the O-antigen and then transported across the periplasm by a transenvelope device, the Lpt protein machinery, composed in *E. coli* by seven essential proteins (LptABCDEFG) (reviewed by [6], [7]) (Figure 1). At the IM, the LptBFG complex constitutes an ABC transporter that provides the energy for LPS transport. LptC is a small bitopic protein [8] that resides in the IM and interacts with the LptBFG complex [9] and with the periplasmic protein LptA [10] [11]. LptA is thought to transfer LPS to the LptDE protein complex of the OM. Thus, LptA is the periplasmic protein that connects the IM Lpt components to the OM LPS translocon (LptD and LptE), which ensures the assembly of LPS at the cell surface [12]–[14]. The Lpt machinery appears to operate as a single device as depletion of any Lpt component leads to common phenotypes that includes the appearance of an anomalous LPS form decorated by repeating units of colanic acid [8], [15], and in such depleted strains the majority of *de novo* synthesised LPS accumulates in a novel membrane fraction (hIM) with higher density than the IM [8]. The process by which hydrophobic LPS is transported across the periplasm to the cell surface is not fully understood. The current model postulates that the Lpt proteins, through homologous domains interactions, create a transenvelope bridge that connects IM and OM [14], [16], thus forming a continuous channel through which LPS is moved to the cell surface [17].

The OM is an essential structure for bacterial survival and the first site of interaction with the mammalian host [18]; mutants defective in OM biogenesis typically display alterations of the OM permeability barrier properties [3]. The crucial role of this structure is highlighted by the fact that in *E. coli* at least five different pathways (Bae, Cpx, Psp, Rcs and σ^E^) constitute signaling systems that detect and respond to alterations of the bacterial envelope [19]–[22]. These pathways regulate expression of complementary functions whose discrete contributions are integrated to mount a full adaptive response [23].

In this work we sought to analyze on a global level the response of *E. coli* cells to a severe OM biogenesis defect, namely the block of transport of LPS upon LptC depletion, to investigate on the cell response to an OM stress. To this purpose we used a proteomic approach based on two-dimensional chromatography coupled to tandem mass spectrometry (2DC-MS/MS), called MudPIT (Multidimensional Protein Identification Technology) [24] to monitor the cell envelope protein content of an arabinose dependent *lptC* conditional mutant [8] grown under permissive and non permissive conditions. Our results highlight pathways and strategies adopted by *E. coli* cells to respond to severe OM biogenesis defects.

## Materials and Methods

### Bacterial strains and growth conditions

The bacterial strains used in this study are derivatives of AM604 (MC4100 ara^+^; [25]. FL905 (AM604 Φ(*kan araC araBp-lptC*)*1*) is a conditional arabinose dependent mutant carrying *lptC* under the control of *araBp* promoter [8]. Bacteria were grown in LD medium [26] at 37°C. When required, 0.2% (w/v) L-arabinose (as an inducer of the *araBp* promoter), and 25 µg/ml kanamycin, were added. The strain PS200 (MC4100 *ara^+^ asmA-SPA::kan*) was obtained by moving the *asmA-SPA::kan* allele from CAG64009 to AM604 by P1 transduction using standard procedures [8]. Subsequently, the *kan* cassette was removed from PS200 by pCP20-encoded Flp recombinase [27], generating the mutant PS201. The removal of the *kan* genes was verified by colony PCR. Finally, to construct strain PS202 [AM604 Φ(*kan araC araBp-lptC*)*1 asmA*-*SPA*], the *kan araC araBp-lptC* allele was moved from FL905 (AM604 Φ(*kan araC araBp-lptC*)*1*) into PS201 byP1 transduction and selecting on media containing kanamycin and 0.2% arabinose.

Transductions were verified by colony PCR and by immunoblotting on whole cell extract using anti-FLAG M2 antibodies (Sigma-Aldrich Inc., St.Louis, MO, USA).

### Whole membrane proteins extraction

AM604 and FL905 cells were grown in LD supplemented with 0,2% arabinose up to OD600 = 0.2 at 37°C. Cells were then harvested, washed in LD, diluted five hundredfold (in fresh medium with or without 0,2% arabinose) and incubated with aeration at 37°C. After 330 minutes, 125 OD of cell cultures were harvested, re-suspended in 3 ml of 10 mM Tris (pH = 8.0), 1 mM EDTA, 1 mM PMSF, 0,2 mg/ml lisozyme and incubated on ice. After 30 minutes, 0,2 mg/ml DNAse was added and cells were disrupted by sonication (6 cycles of 10 seconds at 20% amplitude). The cleared lysates were then subjected to centrifugation at 100,000×g for 60 min at 4°C. Pellets, that contain whole cell membranes, were re-suspended in MilliQ water and lyophilized, or analysed by western blotting using anti-LptC, anti-LptE, anti-LptB, anti-AcrB or anti-FLAG M2 antibodies (Sigma-Aldrich Inc., St.Louis, MO, USA).

### Cell fractionation

AM604 and FL905 cells were grown as described above. Cells were chilled in ice and harvested by centrifugation. IM and OM were separated by discontinuous sucrose density gradient centrifugation of a total membrane fraction obtained by spheroplast lysis as described previously [28]. Step gradients were prepared by layering 2 ml each of 50, 45, 40, 35, and 30% (wt/vol) sucrose solutions over a 55% sucrose cushion (0.5 ml). Fractions (300 µl) were collected from the top of the gradient, 50 µl of each fraction was assayed for NADH oxidase activity [28]. The total protein concentration of each fraction was determined by the Bradford assay (Thermo Fisher Scientific Inc. Waltham, MA) as recommended by the manufacturer. The protein profiles of OmpC, OmpF, and OmpA across the gradient were estimated by separating 20 to 40 µl of each fraction on 12.5% SDS-PAGE and by staining the gels with Coomassie blue. Fractions corresponding to IM, hIM, and OM were lyophilized and analyzed.

For the subcellular localization of AsmA-SPA, PS201 [AM604 *asmA*-*SPA*] and PS202 [AM604 Φ(*kan araC araBp-lptC*)*1 asmA*-*SPA*] cells were grown in LD or LD with 0.2% arabinose up to OD_600_ = 0.2. Cells were then harvested, washed in LD, diluted three hundredfold (in fresh medium with or without 0,2% arabinose) and incubated with aeration at 37°C. After 280 minutes, 125 OD of cell cultures were harvested. IM and OM were separated by discontinuous sucrose density gradient centrifugation of total membranes as described above. 20 µl of each fraction collected from the gradient were separated on 10% SDS-PAGE followed by immunoblot analysis using monoclonal anti-FLAG M2 antibodies (Sigma-Aldrich Inc., St.Louis, MO, USA) to detect AsmA-SPA.The 55 kDa IM protein that is detected by anti-LptD antibodies and the OM protein LamB were used as IM and OM markers, respectively [8].

### Proteomic Analysis

Lyophilized samples were first resuspended in 0.1 M ammonium bicarbonate, pH 8.0, and then treated with RapiGest SF (Waters Corporation, Milford, MA, USA) at the final concentration of 0.2% (w/v). After incubation at 100°C for 5 min, the samples were cooled to room temperature and digested with trypsin (Sequencing Grade Modified Trypsin, Promega, Madison, WI, USA). Initially, trypsin was added to mixtures at an enzyme/substrate ratio of about 1∶50 (w/w) and incubated at 37°C overnight, then another aliquot of enzyme was added at an enzyme/substrate ratio of 1∶100 (w/w) and the samples were incubated at 37°C for 4 hours.

The enzymatic reactions were chemically stopped by acidification with TFA 0.5% (Sigma-Aldrich Inc., St.Louis, MO, USA), incubation at 37°C for 45 min and centrifugation at 13,000×g for 10 min in order to remove hydrolytic RapiGest SF by-products.

Before MudPIT analysis, samples were desalted by PepClean C-18 spin columns (Pierce Biothecnology Inc., Rockford, IL, USA), concentrated in a SpeedVac (Savant Instruments Farmingdale, NY, USA) at 60°C and finally resuspended in 0.1% formic acid (Sigma-Aldrich Inc., St.Louis, MO, USA).

Trypisn-digested peptides were analyzed by two dimensional micro-liquid chromatography coupled to ion trap mass spectrometry (Multidimensional Protein Identification Technology (MudPIT)) using ProteomeX-2 configuration (Thermo Electron Corporation, San Josè, CA, USA) [29]. 10 µl of the digested peptide mixtures were loaded by means of an autosampler (Suveyor AS Thermo) onto a strong cation exchange column (BioBasic-SCX, 0.32 i.d.×100 mm, 5 µm, Thermo Electron Corporation, Bellofonte, PA, USA) and then eluted using eight steps of increasing ammonium chloride concentration (0, 20, 40, 80, 120, 200, 400, 700 mM). Eluted peptides, obtained by each salt steps, were at first captured in turn onto two peptide traps (Zorbax 300 SB C-18, 5 µm, 0.3 id×5 mm, Agilent technologies, Santa Clara, CA, USA) mounted on a 10-port valve, for concentration and desalting, and subsequently loaded on a reversed phase C-18 column (BioBasic-18, 0.180 i.d.×100 mm, 5 µm, Thermo Electron Corporation, Bellofonte, PA, USA) for separation with an acetonitrile gradient. The gradient profile was: 5–10% eluent B in 5 min, 10–40% eluent B in 40 min, 40–80% eluent B in 8 min, 80–95% eluent B in 3 min, 95% eluent B for 10 min, 95–5% eluent B in 4 min and 5% eluent B for 15 min (eluent A, 0.1% formic acid in water; eluent B, 0.1% formic acid in acetonitrile). The flow rate was 100 µl/min slit in order to achieve a final flux of 1 µl/min.

The peptides eluted from the C-18 column were directly analysed with an ion trap mass spectrometer (LCQ Deca XP plus) equipped with a nano electrospray ionization source (nano-ESI) (Thermo Finnigan Corp., San Josè, CA, USA). The heated capillary was held at 185°C; full mass spectra were acquired in positive mode and over a 400–2000 *m/z* range, followed by three MS/MS events sequentially generated in a data-dependent manner on the first, second and third most-intense ions selected from the full MS spectrum, using dynamic exclusion for MS/MS analysis (collision energy 35%).

The experimental mass spectra produced by MudPIT analyses were correlated to tryptic peptide sequences by comparing with theoretical mass spectra, obtained by *in silico* digestion of *Escherichia coli* protein database downloaded from the NCBI website (www.ncbi.nlm.nih.gov/Ftp/index.htlm). Data processing was performed using the 3.3.1. Bioworks version, based on SEQUEST algorithm (University of Washington,licensed to Thermo Finnigan Corp., San Josè, CA, USA), and the following parameters: Xcorr scores greater than 1.5 for singly charged peptide ions and 2.0 and 2.5 for doubly and triply charged ions, respectively, the peptide probability ≤0.001 and the protein consensus score value ≥10. These filters guaranteed that the resulting proteins have a probability value *p*≤0.001.

Data were treated with an in-house algorithm called MAProMa [30] (Multidimensional Algorithm Protein Map), in particular a tool of MAProMa permits the comparison of the protein list obtain from the analysis of the samples.

Two biological replicates for the three samples of total membrane and for the samples obtained from the fractionation of the membranes were analysed and for each of them two technical replicates were made.

The reproducibility of the method was evaluated as described in our previous work [31].

Proteins with significant differences in level, were identified by other two tools of MAProMA: DAve (Differential Average) and DCI (Differential Coefficient Index) [32]. These two algorithms are based on score values assigned by SEQUEST software to each identified protein in samples to be compared. Specifically, DAve is an index of the relative ratio between control and mutant and DCI is an index to evaluate the absolute variation of score value of each protein. Briefly, using MAProMA each identified protein in the two samples were aligned and then DAve and DCI indexes were calculated for all proteins. The threshold values imposed were very stringent: DAve >0.4 and DAve<–0.4, DCI>400 and DCI<–400. To increase the confidence, it is necessary that both indexes, DAve and DCI, satisfy these thresholds.

## Results and Discussion

### Proteomic profiles of cell envelope upon LptC depletion

**Figure 2:**
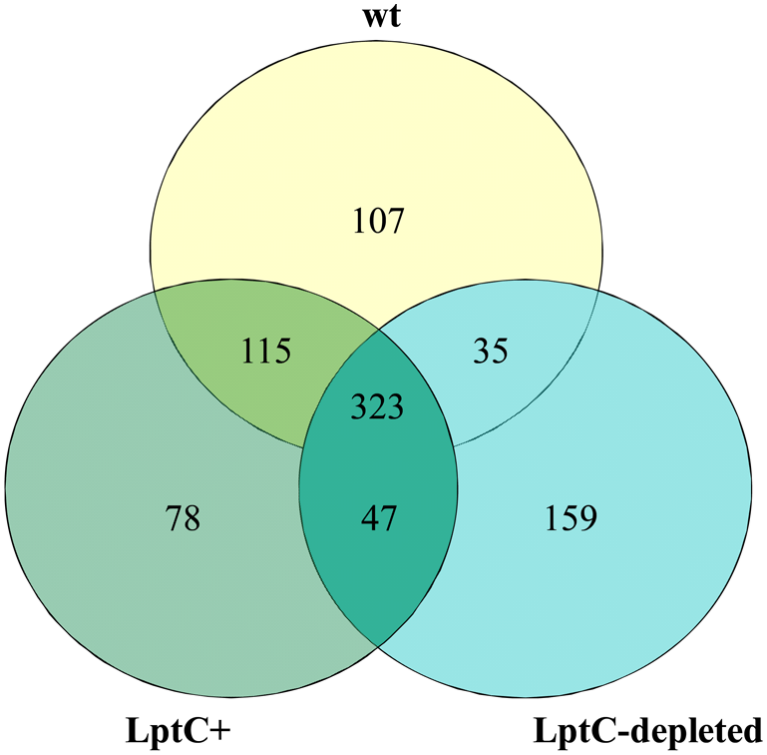
Venn diagram of proteins distribution across strains and growth conditions analysed. Proteins are identified from total membrane samples. wt, (PS201); LptC^+^ (PS202, *araBp-lptC*) grown under permissive condition (0,2% arabinose); LptC-depleted, PS202 grown under non permissive condition (without arabinose).

To understand how cells respond to severe OM damage, we compared the cell envelope protein content of the conditional mutant FL905 grown under permissive and non-permissive conditions for LPS transport to the cell surface. In FL905 *lptC*, which encodes a component of the LPS transport (Lpt) machinery, is under control of the arabinose inducible *araBp* promoter and, upon LptC depletion in the absence of arabinose, LPS transport is blocked [8]. FL905 was grown under permissive (with 0.2% arabinose, FL905+A) and non-permissive (without arabinose, FL905–A) conditions and membrane proteins were extracted and analysed by MudPIT. As a control, the isogenic *lpt* ^+^ strain AM604 [25] was used. MudPIT has been extensively used for protein identification and characterization and provides a significant improvement over gel-based analysis, as it represents a fully automated and high-throughput technology. By this approach we identified about 300 proteins in each sample (Table S1). In particular, a total of 864 proteins were identified in the three samples of total membrane analysed in replicate and among these, 323 were detected in all the three samples. 115 were detected in AM604 and FL905+A and 35 in AM604 and FL905–A, whereas 47 were shared between FL905+A and FL905–A. 107, 78 and 159 unique proteins were exclusively detected in AM604 strain, in FL905+A and in FL905–A, respectively (Figure 2).

Using a in-house software called MAProMA [30], the protein lists identified in the three samples of total membranes were plotted on a 2-D map according to the theoretical MW and pI of identified proteins. Figure S1 reports as an example the 2D-map of the proteins obtained by the analysis of the FL905–A mutant membranome.

Using DAve and DCI algorithms of MAProMa software, the relative protein abundance, of AM604 and FL905 grown under permissive conditions, was evaluated by means of a label-free quantitative approach. DAve and DCI were calculated for each pairwise comparison. Only the proteins that exceed the set threshold values were considered. In FL905 the *araBp* promoter drives the expression of *lptCAB* [8]. Thus as expected we found increased abundance of LptC and the IM associated protein LptB in FL905 as compared with the wild type AM604 control. On the contrary, the periplasmic LptA protein was not detected in the membrane fraction of either strain. It is worth to mention that in the *lptC* depletion strain grown under non-permissive conditions *lptAB* are expressed from a σ^E^ dependent promoter located within the *lptC* coding sequence [33]. With the exception of proteins involved in arabinose metabolism (Table S1) no other significant differences in protein relative abundance were detected between AM604 and FL905+A. For this reason the latter was used as the reference condition in all comparisons with FL905 grown without arabinose (data not shown).

The comparative analysis of the cell envelope protein profile of FL905+A *versus* FL905–A cells showed 123 proteins differentially represented (Table S2). These proteins are related to different cellular pathways that collectively give a snapshot on cellular pathways modulated by *E. coli* cells to respond to severe OM biogenesis defects. As reported in the following paragraphs the functions assigned to the proteins differentially expressed fall in two main pathways: cell envelope biogenesis/remodeling, and protein transport/assembly at the OM. In addition we observed lower abundance for several proteins with a function in (i) central metabolism, (ii) translational apparatus (iii) protein folding/degradation. (Table S2). As lower abundance of these proteins may be correlated with growth arrest imposed by block of LPS transport [8], [10], [15], [34], these functions will not be further discussed. Interestingly the expression of many functions identified by our analysis is under the control of signalling systems (Bae, Cpx, Rcs, and σ^E^) that in *E. coli* detect and respond to alterations of the bacterial envelope [19]–[22].

Previous work [8], [15] showed that mutant cells depleted of any Lpt component exhibit strikingly similar multilayer membranous bodies in the periplasm (hIM). In this contest, to understand the nature of the hIM and the changes that occur at the level of bacterial membrane in order to restore homeostasis, we used discontinuous sucrose density gradient centrifugation to fractionate IM, OM and hIM from FL905+A and FL905–A and we analysed the protein profile of each fraction by MudPIT (Table S1).

Below the main pathways showing different protein levels when comparing FL905+A and FL905–A are discussed.

### Cell envelope biogenesis/remodeling

#### Peptidoglycan synthesis and cell division

Lpt proteins depletion leads to growth arrest and in the late phase of depletion cells show mostly short filaments [8], [10]. In line with this phenotype we found that the level of many proteins implicated in peptidoglycan biosynthesis decreases (Table 1). The transglycosylase MurG, the transpeptidase MrdA (PBP2), peptidoglycan hydrolases AmiA, EmtA, and MltB all have a role during cell elongation [35], [36] and show decreased abundance in FL905-A. The same applies to LpoA and LpoB OM lipoproteins that modulate the activity of transpeptidases involved in cell elongation [37]. TolB, a periplasmic protein that binds peptidoglycan via the Pal lipoprotein [36], [38] and required for OM invagination also shows a decreased level whose significance is difficult to explain. On the contrary the level of proteins involved in peptidoglycan remodeling and cell division appears to increase in LptC depleted cells (Table 1). In fact we found increased abundance for DacA (PBP5) and DacC (PBP6) carboxypeptidases that remove terminal D-alanine residues from pentapeptide side chains thus preventing those side chains from serving as donors for transpeptidation [39]. Also the level of PBP1B a major peptidoglycan synthase involved in cell division [39], FtsZ the key player at the division machinery apparatus [40] and ZipA a protein interacting with FtsZ and required for preseptal peptidoglycan synthesis [41], increases upon LptC depletion.

**Table 1:** Envelope proteins exhibiting a significant variation upon LptC depletion: peptidoglycan synthesis/remodeling and cell division.

Category^a^	GI Accession^b^	Protein	Activity	DAve^c^
**Peptidoglycan synthesis**	16128083	MurG	Precursors synthesis - GTases	0,50
	16128142	PBP1B	Synthesis - GTases and DD-TPases	−1,23
	16128615	PBP5	Regulation of structure-DD-CPases	−0,58
	16128618	PBP2	Synthesis - DD-TPases	2,00
	16128807	PBP6	Regulation of structure-DD-CPases	−2,00
	16129068	LpoB	Regulation of synthesis-Synthase activators	0,50
	16129156	EmtA	Hydrolysis - Lytic transglycosylases	1,00
	16129736	MipA	Synthesis -Scaffolding protein	0,66
	16130360	AmiA	Hydrolysis - Amidases	1,19
	16130608	MltB	Hydrolysis - Lytic transglycosylases	0,85
	16131039	LpoA	Regulation of synthesis - Synthase activators	0,65
**Cell division**	16128088	FtsZ	Cytosckeletal structure	−1,26
	16128715	TolB	Outer membrane invagination	0,46
	16130338	ZipA	Early association with Z ring	−0,76

a inferred from ecocyc.org.

b NCBI accession number.

c DAve value ranges from −2 and +2; positive value for DAve indicates that the protein is more abundant in LptC+ (grown with 0.2% arabinose); negative value for DAve indicates that the protein is more abundant in LptC-depleted (grown without arabinose).

Thus it appears that in LptC depleted cells the synthesis of peptidoglycan is inhibited and that the arrest of cell wall growth shifts LptC depleted bacteria to the cell division program as suggested by the increased level of functions implicated in septation; this is in line with the notion that bacteria must somehow coordinate peptidoglycan synthesis with OM assembly. The recent discovery of OM lipoproteins LpoA and LpoB as modulators of PBPs activity suggests that they may play a crucial role in coupling OM biogenesis with PG synthesis [37]. Interestingly we found that the level of both lipoproteins decreases in LptC depleted cells further strengthening the idea that peptidoglycan synthesis is also controlled from outside of the sacculus.

#### Known/putative proteins involved in LPS/OMP biogenesis

**Figure 3:**
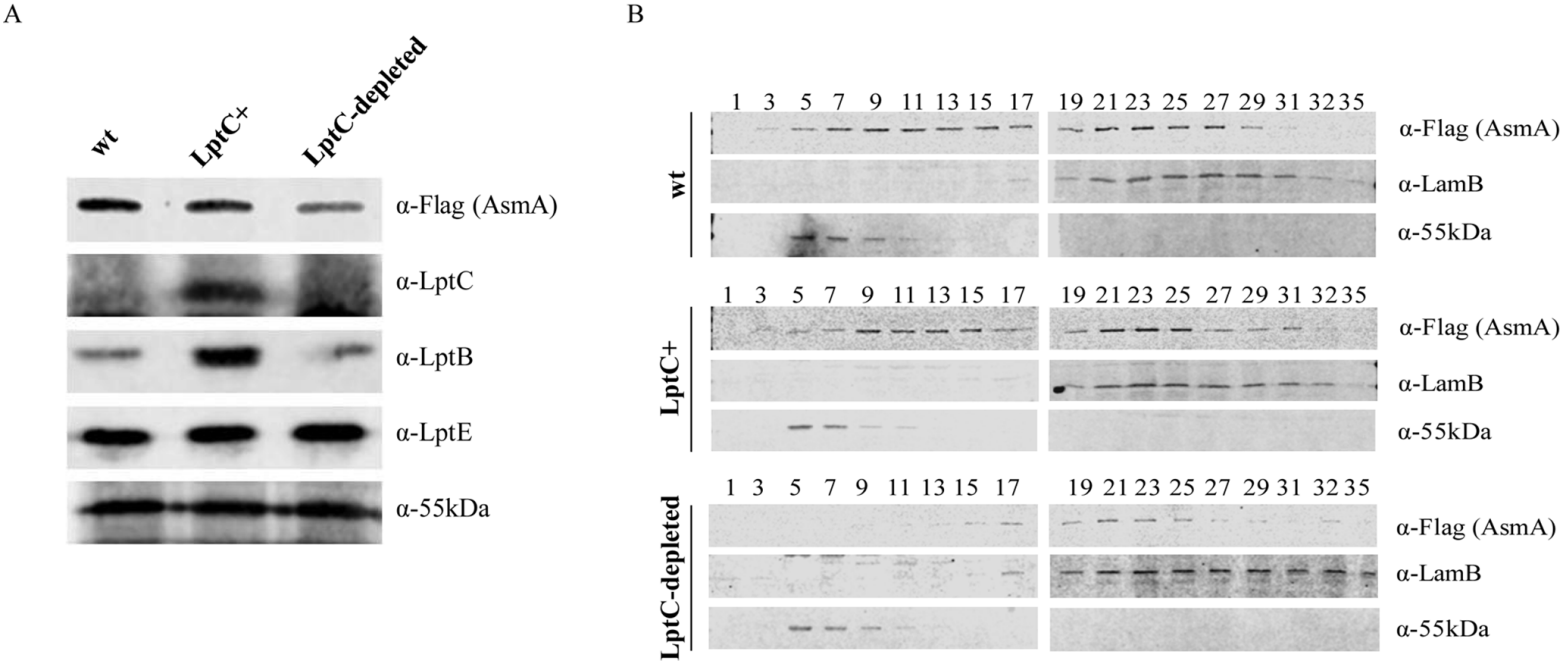
AsmA protein abundance and subcellular localization. PS201 (*asmA-SPA lptC^+^*) and PS202 (*asmA-SPA araBp-lptC*) cells were grown with or without arabinose as indicated. Total membrane protein extracts prepared as described in Materials and Methods were analysed by immunoblotting (panel A) or fractionated by sucrose density gradient (panel B). A) 10 µg of total membrane proteins were loaded in each lane. 55-kDa protein was used as loading control. B) Fractions were collected from the top of the gradient and immunoblotted using antibodies recognizing the 55-kDa protein as IM marker, LamB as OM marker. α-Flag antibodies were used to detect AsmA-SPA protein. wt, PS201; LptC^+^, PS202 (*araBp-lptC*) grown under permissive condition (with 0,2% arabinose); LptC-depleted, PS202 grown under non permissive condition (without arabinose).

Upon LptC depletion the level of proteins involved in LPS biogenesis (LptD, MsbA, WbbK and WbbI) decreases (Table 2). The decreased level of the OM LptD protein (Table 2) is in line with our previous observation that the steady state level of LptA is affected by depletion of LptC or LptD/E [11]. In fact depletion of LptC or LptD removes the IM and OM docking site, respectively, of LptA resulting in LptA degradation [11]. Assembly of LptD requires lipoprotein LptE [42] and the Bam complex [43]. LptE was not detectable even in samples grown under permissive conditions, possibly because it resides within the LptD β-barrel [13] and therefore it may be protected from trypsin degradation. However, LptE level does not change upon LptC depletion (Figure 3A) in line with previous data [11]. LptF, and LptG were not detectable even in samples grown under permissive conditions due to their very low abundance in the cell [15]. Interestingly our data show a decreased level of BamA and BamD, two members of the Bam machinery (see Protein transport/assembly paragraph below). The lower LptD level may thus be the result of a less efficient assembly at the OM. Recently it has been shown that the periplasmic protease BepA, whose expression is regulated by σ^E^, specifically degrades LptD when it fails to form the OM LPS translocon [44]. As an alternative hypothesis we suggest that LptC depletion might affect the formation of a functional OM translocon that undergoes degradation by BepA [15]. Overall, from our data it is not possible to discriminate whether decreased LptD level is the result of lower synthesis, less efficient assembly or degradation of non-functional translocon.

**Table 2:** Envelope proteins exhibiting a significant variation upon LptC depletion: cell envelope biogenesis.

Category^a^	GI Accession^b^	Protein	Activity	DAve^c^
**Known proteins involved in LPS biogenesis**	16128048	LptD	Transporter	0,43
	16128881	MsbA	Transporter	0,80
	16129972	WbbK	Biosynthesis-O antigen	1,40
	16129974	WbbI	Biosynthesis-O antigen	0,85
**Putative proteins involved in OM biogenesis**	16130004	AsmA	Assembly of OM proteins?	2,00
**Functions involved in OM homeostasis**	16128173	FabZ	Biosynthesis - β-hydroxyacyl-ACP dehydratases	−1,19
	16128178	AccA	Biosynthesis - Acetyl-CoA carboxylase A	−0,54
	16128757	YbhO	Biosynthesis - Cardiolipin synthase	−2,00
	16129985	WcaK	Synthesis - glycosyl transferase	−2,00
	16129990	WcaI	Synthesis - glycosyl transferase	−2,00
	16129993	Gmd	Synthesis - GDP-mannose 4,6-dehydratase	−2,00
	16129997	WcaC	Synthesis - glycosyl transferase	−2,00
	16130002	Wza	Export - capsular polysaccharide	−2,00
	16130251	AccD	Biosynthesis - Acetyl-CoA carboxylase D	−2,00
	16130740	Aas	Biosynthesis - Hydroxycinnamate-CoA ligase	1,19
	16131083	MlaD	Transporter - Phospholipids	−1,19
	16131084	MlaE	Transporter - Phospholipids	−2,00
	16131641	WzzE	Regulator of O length	−0,50
	16131985	Psd	Biosynthesis - Phosphatidylserine decarboxylase	−1,13
	33347613	WzzB	Regulator of O length	−0,99
	33347615	Wzc	Export - capsular polysaccharide	−2,00
	33347817	PlsB	Biosynthesis - Glycerol-3-phosphate acyltransferase	−0,77
**Protein membrane turnover**	16128154	DegP	Protease - Periplasmic serine protease	−1,52
	16131068	FtsH	Protease - ATP-dependent metalloprotease	−1,35
	16131996	HflK	Regulator of FtsH	−1,55
	16131997	HflC	Regulator of FtsH	−1,41

a inferred from ecocyc.org.

b NCBI accession number.

c DAve value ranges from −2 and +2; positive value for DAve indicat that the protein is more abundant in LptC+ (grown with 0.2% arabinose); negative value for DAve indicates that the protein is more abundant in LptC-depleted (grown without arabinose).

MsbA is the essential ABC transporter implicated in flipping LPS across the IM [4], [5] and operates in the LPS export pathway just upstream the Lpt protein machinery (Table 2). No physical interactions have been detected so far between MsbA and the Lpt machinery. Our data for the first time point to a functional interaction between the two systems and suggest that arrest of LPS transport and its accumulation at the periplamic face of the IM somehow affects MsbA level.

We found that the level of WbbI (galactofuranosyl transferase) and WbbK (glucosyl transferase) decreased upon LptC depletion (Table 2). The *wbbI* and *wbbK* genes map at a locus encoding genes involved in O-antigen subunits and other exopolysaccharides (including colanic acid) biosynthesis [2]; in particular, *wbbIJKL* genes have been implicated in biosynthesis of O16 antigen subunit [45]. *E. coli* K12 LPS, however, is lacking the O-antigen as *wbbL*, which codes for a rhamnosyltransferase, is interrupted by an IS*5* insertion [46]. It thus appears that in strain FL905 (an *E. coli* K12 derivative) the loss of *wbbL* does not prevents the expression of other *wbb* genes, whereas the block of LPS transport affects *wbb* genes expression resulting in lower levels of WbbI and WbbK.

Finally, in LptC depleted cells the level of AsmA, a non-essential IM protein of unknown function (Table 2, Figure 3A) for which a possible role in OM biogenesis has been previously proposed [47], decreases. The role of AsmA in OM biogenesis stems from the observation that a mutant *asmA* allele is able to correct the assembly defect of mutated OmpC and OmpF proteins [48], [49]. In agreement with a putative role in OM biogenesis, *asmA* null mutants show increased sensitivity to hydrophobic antibiotics and a decreased LPS level [47]. In contrast to previous reports [47] we found that in wild type and in non LptC-depleted cells AsmA localizes in both the IM and OM (Table 3), a typical behaviour of membrane fusion proteins (MFP) that function in bridging IM and OM in Gram-negative bacteria [50]. Interestingly, in LptC depleted cells not only the level of AsmA decreased but also its subcellular localization changed as the protein disappeared from the IM and localized at the hIM and OM in LptC depleted cells (Table 3 and Figure 3B). Our data support the role of AsmA in OM biogenesis highlighting a functional link between the assembly of OM proteins and that of LPS as the correct balance of both OM components is required to build a functional OM.

**Table 3:** AsmA level in membrane fractions.

Strain/condition	IM		hIM		OM	
	Score^a^	Hits^b^	Score^a^	Hits^b^	Score^a^	Hits^b^
**wt**	40,25	7	nd	nd	50,26	9
**LptC+**	30,23	4	nd	nd	60,21	8
**LptC-depleted**	0	0	50,30	7	30,33	3

a SEQUEST score value, related to the confidence of identification.

b number of identified peptides.

#### Functions involved in OM biogenesis/remodelling

Several proteins (WzzE, WzzB, WcaC, WcaK, Gmd, WcaI, Wza, Wzc) belonging to the 19 genes *wca* (*cps*) cluster and implicated in colanic acid biosynthesis [51], [52] showed increased abundance in LptC depleted cells (Table 2). This finding is in agreement with our previous observation that depletion of any Lpt protein leads to the production of LPS decorated by colanic acid, indeed this modification is diagnostic of defects in LPS transport occurring downstream of MsbA mediated lipid A-core flipping across IM [8]. Interestingly, *Salmonella enterica* mutants defective in LPS transport due to mutations in *lptC* also show an altered LPS profile [53]. Although the chemical nature of such modification is not known it is reminiscent of the high molecular weight ladder observed upon Lpt proteins depletion. Colanic acid is a cell surface polysaccharide synthesised by enteric bacteria in response to envelope damaging conditions such as osmotic, acid and cold stresses [21], [54]. Expression of *cps* cluster is controlled by the Rcs proteins that constitute a complex phosphorelay system known to extend well beyond regulation of colanic acid synthesis [21]. We recently showed that *E. coli* cells treated with ammonium metavanadate, a phosphatase inhibitor known to induce covalent modification of lipid A [55], also produce LPS decorated by colanic acid [33]. The signals that activate such pathways are not well known, however our data support the idea that a specific “LPS” stress may induce the Rcs system therefore activating functions needed for surface remodelling.

In agreement with the notion that block of LPS export pathway results in migration of phospholipids in the outer leaflet of the OM, we found that the level of two components of Mla pathway, MlaD and MlaE, increases upon LptC depletion (Table 2). The Mla (Maintenance of OM lipid asymmetry) proteins function as an inter-membrane transport system to prevent surface exposure of phospholipids upon stressful conditions thus maintaining OM asymmetry [56]. Several mutants in LPS biogenesis display increased OM permeability as phospholipids may migrate from the inner to the external leaflet of the OM thus generating locally symmetric bilayer rafts freely permeable to hydrophobic compounds [57]. As the Mla system appears to function by retrograde trafficking of phospholipids from the OM to the IM, the increased level of MlaD and MlaE proteins upon LptC depletion might be needed to maintain lipid asymmetry and therefore OM homeostasis. Our data provide the first functional connection between the LPS export and the OM phospholipid removal pathways.

Proteins implicated in *de novo* fatty acids (AccD, AccA, FabZ) and in phospholipids biosynthesis (PlsB, Psd) (Table 2) also show an increased level in LptC depleted cells. AccA and AccD constitute the heterodimeric carboxyltransferase involved in the first reaction of fatty acid synthesis. FabZ is an R-3 hydroxyacyl-ACP dehydrase which provides precursors for phospholipids synthesis [58]. Interestingly, FabZ shares the substrate R-3 hydroxyacyl-ACP with LpxA and LpxD two enzymes involved in lipid A biosynthesis [59] and is therefore a key enzyme in controlling phospholipids and LPS synthesis. Our data suggest that the modulation of levels of FabZ which competes with LpxA and LpxD for the shared precursor, can be a strategy to shift the synthesis towards the production of phospholipids thus limiting lipid A synthesis. Indeed in LptC depleted cells LPS can not be inserted in the outer leaflet of the OM and consequently cells need to fill the “void” with phospholipids. On the other hand, the increased level of PlsB and Psd (Table 2), which are involved in phosphatidlyethanolamine (PE) synthesis [60] suggests that increased synthesis of this specific phospholipid may help cells upon severe cell envelope damage. In fact surface exposed phosphatidlyethanolamine (PE) is the substrate of the inducible EptA enzyme [61] that removes phosphoethanolamine from its donor substrate (PE) and transfers it to the 1-phophate group of lipid A. Such modification has been detected in lipid A of several mutants defective in LPS transport [13], [15] and contributes to polymixin resistance in several organisms such as *Helicobacter pylori* [62], *Neisseria meningitidis* [63] and *Campylobacter jejuni* [64]. Lipid A modifications by EptA are thought to stabilize and/or balance the surface electrostatics of the OM and can be thus considered an additional mechanism to restore the OM barrier function when LPS transport is defective. It is thus possible that in LptC depleted cells PE synthesis increases the donor substrate for lipid A modification as a strategy to restore the OM functionality.

Finally, we found increased level for YhbO, a conserved protein with unknown function that has been implicated in protection against diverse environmental stresses such as oxidative, thermal, osmotic and pH stresses [65]. YhbO increased level upon LptC depletion may also help cells to recover from envelope damaging stresses.

A decreased expression level upon LptC depletion has been observed for Aas (Table 2), an enzyme implicated in membrane phospholipid turnover [66]. The regulation of Aas expression has been poorly investigated and the meaning of our observation is presently not obvious.

#### Membrane protein turnover

The level of FtsH, HflK and HflC proteins increases upon LptC depletion (Table 2). FtsH (also named HflB) is an essential ATP dependent IM protease that interacts with HflK and HflC membrane proteins to form a large holoenzyme complex with a role in quality control and degradation of membrane proteins [67]. FtsH plays also a key role in modulating LPS biosynthesis as it controls by proteolysis the amount of LpxC, which catalyses the first committed reaction in lipid A biosynthesis, and of KdtA, a glycosyltransferase involved in Kdo synthesis [68]. The increased amount of FtsH in LptC depleted cells might lower LPS synthesis as a strategy to cope with the block of its transport and to prevent its accumulation at the IM. Interestingly, in addition to the σ^70^ dependent promoter, a σ^E^ promoter has been predicted upstream the *ftsH* gene [69]. We previously showed that LptC depletion activates the σ^E^ dependent regulon [33]. Our data suggest that the predicted σ^E^ sequence upstream of *ftsH* may represent a functional promoter.

DegP is a periplasmic protein functioning both as a protease and as a chaperone whose expression is induced upon cell envelope stresses [70]. DegP increased level in LptC depleted cells (Table 2) fits with its dual role of protease and chaperone in being able to both degrade irreversibly damaged proteins and to assist/promote folding of partially unfolded periplasmic or OM proteins.

### Protein transport/assembly

Upon LptC depletion we observe a decreased level for BamA and BamD (Table 4) the essential components of the multiprotein machinery responsible for OMP assembly at the OM [43]. LptD, the only known essential OMP, is also a Bam complex substrate. BamA, whose expression is regulated by σ^E^ [71], has recently been shown to be a substrate of the BepA protease when BamA assembly at the OM is compromised [44]. BamD interaction with BamA appears to stabilize the Bam complex [72], [73]; therefore BamD may be an additional BepA substrate or may be degraded by not yet known proteases. The non-essential OM lipoproteins BamB [74] and YiaD instead showed increased abundance (Table 4). YiaD has been identified as multicopy suppressor of a temperature sensitive *bamD* allele [75] and BamB expression is under σ^E^ control [76] possibly explaining their increased level. It thus appears that in Lpt depleted cells growth arrest and damaged envelope both contribute to a general reduction of protein synthesis and therefore to lower level of components of the machinery that insert β-barrel proteins in the OM.

**Table 4:** Envelope proteins exhibiting a significant variation upon LptC depletion: transport/assembly.

Category^a^	GI Accession^b^	Protein	Activity	DAve^c^
**Protein transport**	16128091	SecA	Transporter -Sec Translocation Complex	1,55
	16128170	BamA	Transporter - OMPs	0,54
	16128392	YajC	Transporter -Sec Translocation Complex	0,66
	16128393	SecD	Transporter -Sec Translocation Complex	0,93
	16128394	SecF	Transporter -Sec Translocation Complex	0,66
	16130437	BamB	Transporter - OMPs	−1,19
	16130516	BamD	Transporter - OMPs	0,57
	16131423	YiaD	Transporter - OMPs	−1,42
**Transport**	16128446	AcrA	Membrane fusion protein	−1,63
	16128447	AcrD	Permease	−1,17
	16128896	OmpF	General Bacterial Porin	0,83
	16130014	MdtA	Membrane fusion protein	−1,32

a inferred from ecocyc.org.

b NCBI accession number.

c DAve value ranges from −2 and +2; positive value for DAve indicates that the protein is more abundant in LptC+ (FL905 grown with 0.2% arabinose); negative value for DAve indicates that the protein is more abundant in LptC-depleted (FL905 grown without arabinose).

An important function of the OM is the control of influx and efflux of nutrients and toxic compounds playing an important role in the adaptation to different environmental conditions. Accordingly, in LptC depleted cells where the OM is damaged we observed modulation of the level of proteins that regulate the intracellular influx of toxic compounds. OmpF is a porin with a role in the influx of small molecules [1] whose level decreases. Instead, the level of components of efflux pumps, such as AcrA, AcrB, MdtA increases (Table 4). AcrA, AcrB and MdtA are proteins belonging to multidrug efflux pumps, whose expression is up-regulated in response to envelope-damaging agents [77], [78]. We confirmed by western blotting that AcrB level indeed increases upon LptC depletion (Figure S2).

### Extra-cytoplasmatic stress response

**Figure 4:**
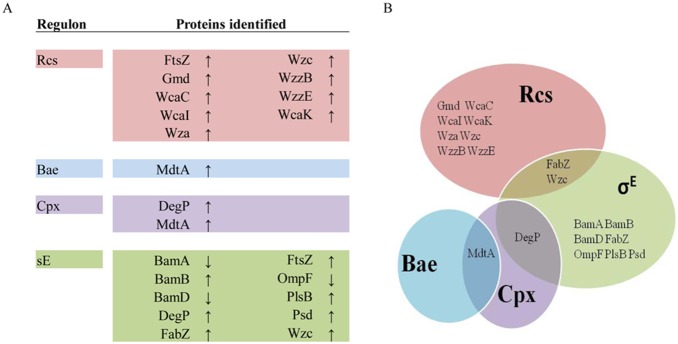
List of proteins belonging to cell envelope stress response pathways whose level changes upon LptC depletion. A. Relative abundance is calculated as the ratio beetween FL905 grown under permissive and non permissive conditions: ↑, increased; ↓, decreased (see Tables 1, 2. 4). B. Venn diagram showing functions whose regulations is shared by multiple envelope signaling systems.

The OM functionality is essential for survival in Gram-negative bacteria and therefore its integrity in *E. coli* is monitored by at least five different but overlapping stress response systems (RpoE, Rcs, BaeR, Cpx, Psp) [79]. In LptC depleted cells the relative abundance of 26 proteins belonging to four such pathways (RpoE, Rcs, BaeR, Cpx) increased and three (out of 10) belonging to RpoE pathway (BamE, BamD, OmpF) decreased (Figure 4A). Interestingly, this list includes most of the functions that have been discussed in the previous paragraphs thus highlighting the importance of such pathways in triggering adaptive responses to OM dysfunction (Figure 4B).

### Proteins of hIM

Depletion of any Lpt proteins results in appearance of a novel membrane fraction with higher density of the IM (hIM) where most of *the novo* synthesised LPS accumulates [8], [10], [15]. To better clarify the nature of such fraction we analysed its proteome. We considered hIM proteins those showing a score higher that 30 (Table S3). Out of the 53 hIM proteins matching the selected parameter 44% are proteins normally localized at the IM, 21% are OM proteins and the remaining fraction is represented by cytoplasmic (26%), periplasmic (2%) and unknown (7%) proteins (Figure S3). Most of the proteins enriched in the hIM fraction are related to transport systems whereas the rest form a miscellaneous group; notably none of the proteins displaying an increased level in LptC depleted cells was found in hIM (Table S3). These data suggest that that hIM is more similar to the IM than the OM and are in line with the hypothesis that hIM corresponds to the abnormal membrane structures visible by electron microscopy in Lpt depleted cells [8], [10], [15] where proteins belonging to different biological processes, unrelated to each other, remain trapped.

## Conclusions

We used the MudPIT technology to analyze the envelope proteome in LptC depleted cells, which experience a severe OM biogenesis defect due to block of LPS transport to the cell surface. The comparative proteomic analysis between LptC depleted and not-depleted cells highlighted strategies adopted by bacteria to maintain OM homeostasis. The envelope proteome of LptC depleted cells displayed higher abundance of functions that collectively may contribute to repair the OM and restore its permeability barrier properties. Such functions include proteins implicated in maintaining OM asymmetry or involved in the synthesis of phospholipids and exopolysaccharides as substrates for lipid A-core modification enzymes. Lipid A modifications are generally not required for growth under laboratory conditions but confer selective advantages, such as resistance to antimicrobial peptides or the ability to evade the innate immune system [80]. Interestingly, we found that the level of several enzymes implicated in peptidoglycan synthesis/remodeling changes in LptC depleted cells. Indeed, our results suggest that when growth of the OM is compromised by block of LPS transport cells switch from the “elongation mode” of peptidoglycan synthesis to the “constrictive mode” to direct cells towards the cell division program. It has been recently shown that in *E. coli* the enlargement of the peptidoglycan layer requires control or activation not only from the inside of the cells but also from outside by proteins associated to the OM [37], [81]. Overall, our data further support the notion that OM biogenesis and PG synthesis are tightly coordinated pathways.

The block of LPS transport result in growth arrest and as a consequence the level of ribosomal and transport proteins as well as many folding factors decreases in LptC depleted cells. Conversely, the level of several IM, periplasmic and OM proteases increases to cope with increased abundance of proteins that are not assembled at the OM. Our data are consistent with the notion that the extracytoplasmic stress response is activated upon block of LPS transport as the expression of many functions implicated in OM biogenesis, OM remodeling, protein folding/degradation showing increased abundance in LptC depleted cells are under the control of the Bae, Cpx, Rcs and σ^E^ signaling systems [23]. Of note is the finding that the level FtsH, an essential IM anchored protease, increases in LptC depleted cells. FtsH is known to control LPS biosynthesis by degrading LpxC the enzyme that catalyzes the first committed step of lipid A biosynthesis [82]. Our data point for the first time to a feed-back control on lipid A synthesis signaled from the external surface of the cell when LPS transport to the OM is compromised. We do not know how such signal may be transduced inside the cell; a possible candidate in such signal transduction pathway is the recently identified YciM IM protein that has been suggested to act in concert with FtsH to regulate synthesis of lipid A [83], [84], [85].

Overall our results show a snapshot of pathways modulated by *E. coli* cells to respond to a severe OM biogenesis defect namely block of LPS transport, that act integrating complementary functions to restore OM functionality.

## Supporting Information

Figure S1 **2D map of all the proteins identified in the analysis of total membrane.** MAProMa software plots all the proteins according to the theoretical pI and MW. A color/shape code is assigned to each protein according to relative SC value. Proteins with SC≥35 are reported as red/circle, proteins with SC<35 and >15 are reported as blue/square, and proteins with SC≤15 are reported as yellow/triangle code. The dashed box indicates the typical pI and MW ranges for 2-DE.
(TIF)

Figure S2 **AcrB protein abundance upon LptC depletion.** PS201 (*asmA-SPA lptC^+^*) and PS202 (*asmA-SPA araBp-lptC*) cells were grown with or without arabinose. Extracts of total membrane proteins prepared as described in Material and Methods were analysed by immunoblotting using anti-AcrB anti-LptC antibodies. An IM 55-kDa protein was used as loading control. 10µg of proteins were loaded in each lane. wt, PS201; LptC^+^, PS202 grown under permissive condition (0,2% arabinose); LptC-depleted, PS202 grown under non permissive condition (without arabinose). The asterisk (*) indicates a band cross reacting with anti-AcrB antibodies.
(TIF)

Figure S3 **Localization of proteins identified in hIM.**
(TIF)

Table S1 **List of proteins identified in AM604, FL905+A and FL905-A (total membrane, inner membrane, outer membrane, heavy inner membrane (hIM)).**
(XLS)

Table S2 **Proteins identified by comparative analysis of the cell envelope proteome of FL905+A versus FL905–A cells.**
(XLSX)

Table S3 **List of proteins identified in the heavy inner membrane (hIM) of FL905–A.**
(XLSX)
