# Supplementary figures and images for: Dissecting Escherichia coli Outer Membrane Biogenesis Using Differential Proteomics
Source: PLoS One. 2014 Jun 26;9(6):e100941. doi: 10.1371/journal.pone.0100941 (PMC4072712; doi:10.1371/journal.pone.0100941)

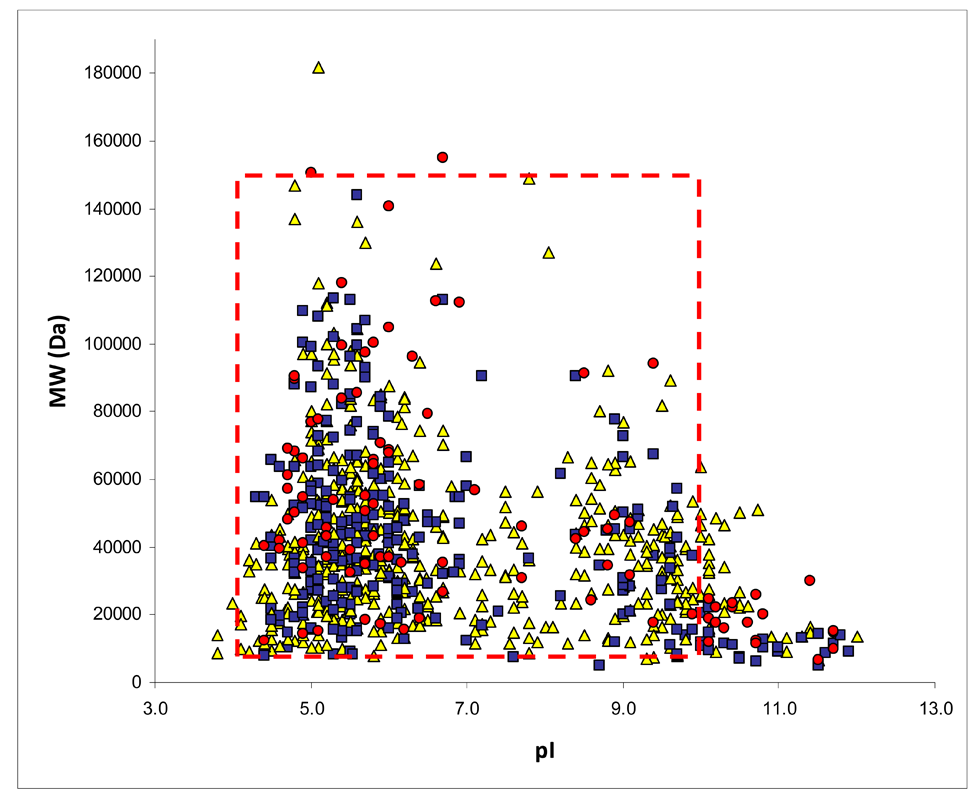

Supplement: Figure S1 — 2D map of all the proteins identified in the analysis of total membrane. MAProMa software plots all the proteins according to the theoretical pI and MW. A color/shape code is assigned to each protein according to relative SC value. Proteins with SC≥35 are reported as red/circle, proteins with SC<35 and >15 are reported as blue/square, and proteins with SC≤15 are reported as yellow/triangle code. The dashed box indicates the typical pI and MW ranges for 2-DE. (TIF) [file pone.0100941.s001.tif]

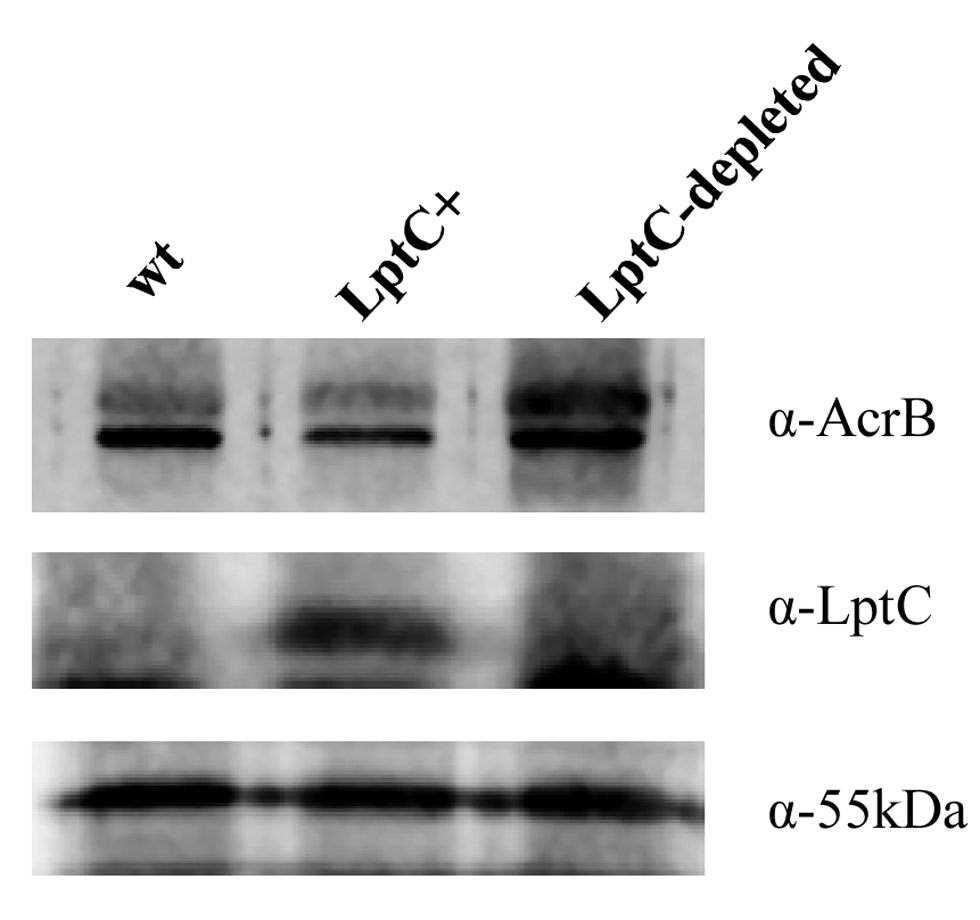

Supplement: Figure S2 — AcrB protein abundance upon LptC depletion. PS201 (asmA-SPA lptC+) and PS202 (asmA-SPA araBp-lptC) cells were grown with or without arabinose. Extracts of total membrane proteins prepared as described in Material and Methods were analysed by immunoblotting using anti-AcrB anti-LptC antibodies. An IM 55-kDa protein was used as loading control. 10µg of proteins were loaded in each lane. wt, PS201; LptC+, PS202 grown under permissive condition (0,2% arabinose); LptC-depleted, PS202 grown under non permissive condition (without arabinose). The asterisk (*) indicates a band cross reacting with anti-AcrB antibodies. (TIF) [file pone.0100941.s002.tif]

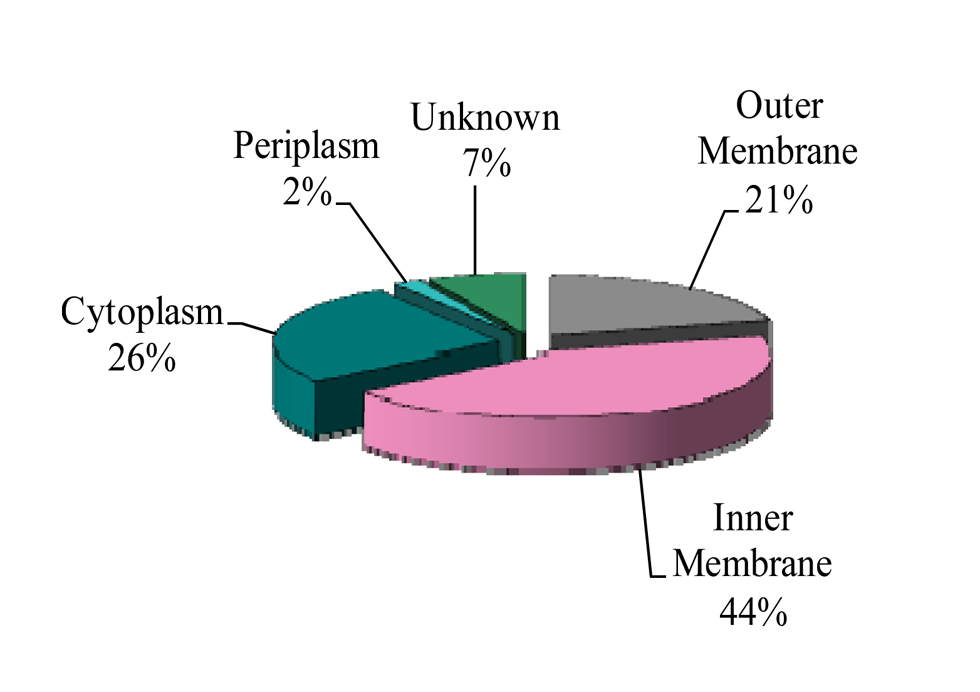

Supplement: Figure S3 — Localization of proteins identified in hIM. (TIF) [file pone.0100941.s003.tif]
